# Decision Rules Derived from Optimal Decision Trees with Hypotheses

**DOI:** 10.3390/e23121641

**Published:** 2021-12-07

**Authors:** Mohammad Azad, Igor Chikalov, Shahid Hussain, Mikhail Moshkov, Beata Zielosko

**Affiliations:** 1 Department of Computer Science, College of Computer and Information Sciences, Jouf University, Sakaka 72441, Saudi Arabia; mmazad@ju.edu.sa; 2 Intel Corporation, 5000 W Chandler Blvd, Chandler, AZ 85226, USA; igor.chikalov@gmail.com; 3 Department of Computer Science, School of Mathematics and Computer Science, Institute of Business Administration, University Road, Karachi 75270, Pakistan; shahidhussain@iba.edu.pk; 4 Computer, Electrical and Mathematical Sciences & Engineering Division, King Abdullah University of Science and Technology (KAUST), Thuwal 23955-6900, Saudi Arabia; 5 Institute of Computer Science, Faculty of Science and Technology, University of Silesia in Katowice, Będzińska 39, 41-200 Sosnowiec, Poland; beata.zielosko@us.edu.pl

**Keywords:** decision rule, decision tree, representation of information, hypothesis

## Abstract

Conventional decision trees use queries each of which is based on one attribute. In this study, we also examine decision trees that handle additional queries based on hypotheses. This kind of query is similar to the equivalence queries considered in exact learning. Earlier, we designed dynamic programming algorithms for the computation of the minimum depth and the minimum number of internal nodes in decision trees that have hypotheses. Modification of these algorithms considered in the present paper permits us to build decision trees with hypotheses that are optimal relative to the depth or relative to the number of the internal nodes. We compare the length and coverage of decision rules extracted from optimal decision trees with hypotheses and decision rules extracted from optimal conventional decision trees to choose the ones that are preferable as a tool for the representation of information. To this end, we conduct computer experiments on various decision tables from the UCI Machine Learning Repository. In addition, we also consider decision tables for randomly generated Boolean functions. The collected results show that the decision rules derived from decision trees with hypotheses in many cases are better than the rules extracted from conventional decision trees.

## 1. Introduction

Decision trees are commonly used as classifiers, as an algorithmic tool for solving various problems, and a means of representing information [1,2,3]. They form a part of statistical learning, which refers to a vast set of tools for understanding data [4]. Conventional decision trees studied in test theory [5], rough set theory [6,7,8], and many other areas of computer science exploit queries based on a single attribute. In [9,10,11,12], we considered decision trees that also exploit queries based on hypotheses. Such decision trees are analogous to the tools that have been analyzed in exact learning [13,14,15], where both membership and equivalence queries are used.

In the present paper, we analyze decision trees with hypotheses as a means for representation of information. We design dynamic programming algorithms to optimize such trees corresponding to two cost functions. For various decision tables, we build optimal decision trees and analyze the length and coverage of decision rules extracted from the constructed trees to study which kinds of decision trees are more suitable for the representation of information.

Let us have a decision table *T* that contains *n* attributes. We can use two types of queries in the decision trees for this table. We can ask about the value of an attribute. As a result, we obtain this value. We can choose an *n*-tuple of possible values of attributes and formulate a hypothesis that it is really the tuple of values of the considered attributes. As a result, we either obtain confirmation of the hypothesis or a counterexample. We call this hypothesis proper if the considered *n*-tuple is a row of the table *T*.

We studied the following five types of decision trees:

1. Using attributes.
2. Using hypotheses.
3. Using both attributes and hypotheses.
4. Using proper hypotheses.
5. Using attributes as well as proper hypotheses.

We analyzed four different cost functions for the decision trees: the depth, the number of realizable nodes, the number of realizable leaf nodes, and the number of internal nodes. We define a node as realizable relative to a given decision table if a computation can pass through this node for at least one row of the considered decision table.

Previously, we proposed a dynamic programming algorithm in [12] for each of these four cost functions. When we give a decision table and a type of decision tree to this algorithm, it returns the minimum cost of a decision tree of a given type for the given table. The results of the computer experiments show that decision trees with hypotheses can have less complexity than conventional decision trees. It means that they can be used as a means for the representation of information.

The present paper has two aims. The first aim is to construct optimal decision trees with hypotheses. We know that such trees can be used for the representation of information (especially decision trees of type 3). However, the algorithms from [12] were designed only to find the complexity of optimal trees. The algorithms for the two cost functions (the depth and the number of internal nodes) can be modified to build optimal decision trees. Unfortunately, we cannot use a similar approach to build optimal decision trees of types 2 and 3 relative to the number of realizable nodes and optimal decision trees of type 2 relative to the number of realizable leaf nodes.

The second aim is to study the length and coverage of decision rules extracted from the optimal decision trees. Decision rules can be considered one of the simplest and most understandable models for the representation of information. Deriving decision rules from decision trees is a well-known approach. We want to confirm that the decision rules derived from decision trees with hypotheses can be better than the rules derived from conventional decision trees.

For computer experiments, we chose eight decision tables from the UCI ML Repository [16] as well as 100 randomly generated Boolean functions that contain *n* variables (n=3,…,6). We constructed optimal (relative to the depth or to the number of internal nodes) decision trees of five types for these tables. Then we analyzed the length and coverage of decision rules extracted from these trees. For a decision tree with hypotheses for some rows of the considered decision table, it can be more than one derived decision rule that covers the row. In this case, for each row we chose the best rule. The results of the computer experiments show that the decision rules derived from the decision trees with hypotheses, in many cases, are better than the ones derived from conventional decision trees.

The novelty of the paper is directly related to its two main contributions: (i) the modification of dynamic programming algorithms described in [12] such that the modified algorithms can now construct optimal decision trees of five types relative to two cost functions and (ii) the experimental confirmation that the decision rules derived from the decision trees with hypotheses can be more suitable for the representation of information than the decision rules derived from conventional decision trees.

To make the paper more understandable, we add to it slightly modified definitions and one algorithm from [12].

We present the remaining parts of the paper as follows: important notions in Section 2 and Section 3, the decision tree optimization based on dynamic programming algorithms in Section 4, Section 5 and Section 6, experimental results in Section 7, and short conclusions in Section 8.

## 2. Decision Tables

We can define a decision table *T* as follows:

- It is a rectangular table that contains *n* (n≥1) columns.
- Its columns are tagged by conditional attributes $f_{1},\ldots ,f_{n}$.
- Its columns’ values are from the set ω={0,1,2,…}.
- Its rows are unique.
- Its rows are tagged by numbers from ω interpreted as decisions.
- Its rows are considered as tuples of values of the conditional attributes.

When a decision table does not have any rows, then we call it an empty table. We define a degenerate table as a decision table which is either empty or has all of its rows tagged by the same decision.

Furthermore, we consider the following notation for *T*:

- F(T) is the set of conditional attributes, i.e., $F\left(T\right)=\left\{f_{1},\ldots ,f_{n}\right\}$.
- D(T) is the set of decisions that are attached to rows.
- $E(T,f_{i})$ is the set of $f_{i}$’s values where $f_{i}\in F\left(T\right)$.
- E(T) is the set of conditional attributes of *T* for which $\left|E(T,f_{i})\right|\;\geq 2$.

Let $S=\{f_{i_{1}}=\delta_{1},\ldots ,f_{i_{m}}=\delta_{m}\}$ be a system of equations where m∈ω, $f_{i_{1}},\ldots ,f_{i_{m}}\in F\left(T\right)$, and $\delta_{1}\in E\left(T,f_{i_{1}}\right),\ldots ,\delta_{m}\in E\left(T,f_{i_{m}}\right)$ (*S* is empty when m=0). We denote by TS the subtable of *T* consisting of all rows of *T* that have values $\delta_{1},\ldots ,\delta_{m}$ when they intersect with the columns $f_{i_{1}},\ldots ,f_{i_{m}}$. Such subtables are called separable subtables of *T*.

## 3. Decision Trees and Rules

In this section, we define notions of decision trees and rules related with a given nonempty decision table *T* that contains *n* conditional attributes $f_{1},\ldots ,f_{n}$. Let us consider the decision trees in connection with two types of queries. The first type of query is to ask the value of an attribute $f_{i}\in F\left(T\right)=\left\{f_{1},\ldots ,f_{n}\right\}$. The answer of this query is from the set $A\left(f_{i}\right)=\left\{\left\{f_{i}=\delta \right\}:\delta \in E\left(T,f_{i}\right)\right\}$. The second type of query is to ask about a hypothesis over *T* in the form of $H=\{f_{1}=\delta_{1},\ldots ,f_{n}=\delta_{n}\}$ where $\delta_{1}\in E\left(T,f_{1}\right),\ldots ,\delta_{n}\in E\left(T,f_{n}\right)$. The answer of this query is from the set $A\left(H\right)=\{H,\left\{f_{1}=\sigma_{1}\right\},\ldots ,\left\{f_{n}=\sigma_{n}\right\}:\sigma_{1}\in E\left(T,f_{1}\right)\setminus \left\{\delta_{1}\right\},...,\sigma_{n}\in E\left(T,f_{n}\right)\setminus \left\{\delta_{n}\right\}\}$. If the answer is *H*, then the hypothesis is true. Other answers are counterexamples. Note that *H* is a proper hypothesis for *T* if $\left(\delta_{1},\ldots ,\delta_{n}\right)$ is a row of the table *T*.

A decision tree over *T* is a tagged finite directed rooted tree, where the following hold:

- We label each leaf node by a number from the set D(T)∪{0}.
- We label each internal node by a hypothesis over *T* or an attribute from the set F(T). In both cases, there is exactly one edge leaving this node for each answer, either from the set A(H) in the case of hypothesis query or from the set $A(f_{i})$ in the case of attribute query, and no other edges exit from this node.

Let us consider a decision tree Γ over *T*. If *v* is a node of Γ, then we define an equation system S(Γ,v) over *T* corresponding to the node *v*. We denote the directed path from the root of Γ to the node *v* as ξ. When ξ does not have any internal nodes, then S(Γ,v) is the empty system. On the other side, if it has internal nodes, then S(Γ,v) is the union of the systems of equation attached to the edges in ξ.

We consider a decision tree Γ over *T* as a decision tree for *T* if, for any node *v* of Γ, the following hold:

- When the node *v* is a leaf node, then the subtable TS(Γ,v) is degenerate and vice versa.
- When *v* is a leaf node and the subtable TS(Γ,v) is empty, then we label the node *v* by the decision 0.
- When *v* is a leaf node and the subtable TS(Γ,v) is nonempty, then we label the node *v* by the decision attached to all rows of TS(Γ,v).

An arbitrary directed path ξ from the root to a leaf node *v* in Γ is called a complete path in Γ. Denote T(ξ)=TS(Γ,v).

The depth of a decision tree Γ is analogous to its time complexity. We denote its depth by h(Γ), which is defined as the maximum number of internal nodes in a complete path in the tree. Similarly, the number of internal nodes in a decision tree Γ (denoted by $L_{w}\left(\Gamma \right)$) is analogous to its space complexity.

Let Γ be a decision tree for *T*, ξ be a complete path in Γ such that T(ξ) is a nonempty table, and the leaf node of the path ξ be tagged with the decision *d*. We now define a system of equations S(ξ). S(ξ) is the empty system in the case of no internal nodes in ξ. Let us assume now that ξ contains at least one internal node. We now transform systems of equations attached to edges leaving internal nodes of ξ. If an edge is tagged with an equation system containing exactly one equation, then we not change this system. Let an edge *e* leaving a internal node *v* be tagged with an equation system containing more than one equation. Then *v* is tagged with a hypothesis *H* and *e* is tagged with the equation system *H*. (Note that if such a node exists, then it is the last internal node in the complete path ξ.) In this case, we remove from the equation system *H* attached to *e* all equations of the kind $f_{j}=\sigma$ such that $f_{j}\notin E\left(TS\left(\Gamma ,v\right)\right)$. Then, we can obtain S(ξ) as the union of new equation systems corresponding to edges in the path ξ. One can show that T(ξ)=TS(ξ).

We correspond to the complete path ξ the decision rule,

$$\underset{f_{i}=\delta \in S\left(\xi \right)}{⋀}\left(f_{i}=\delta \right)\to d.$$

We denote this rule by rule(ξ). The number of equations in the equation system S(ξ) is called the length of the rule rule(ξ) and is denoted l(rule(ξ)). The number of rows in the subtable TS(ξ) is called the coverage of the rule rule(ξ) and is denoted c(rule(ξ)).

Denote Ξ(T,Γ) the set of complete paths ξ in Γ such that the table T(ξ) is nonempty and Rows(T) the set of rows of the decision table *T*. For a row r∈Rows(T), we denote by l(r,T,Γ) the minimum length of a rule rule(ξ) such that ξ∈Ξ(T,Γ) and *r* is a row of the subtable TS(ξ), and we denote by c(r,T,Γ) the maximum coverage of a rule rule(ξ) such that ξ∈Ξ(T,Γ) and *r* is a row of the subtable TS(ξ).

We use the following notation:

$$\begin{array}{ccc} l(T,\Gamma ) & = & \frac{\sum_{r\in Rows(T)}l\left(r,T,\Gamma \right)}{\left|Rows(T)\right|},\\ c(T,\Gamma ) & = & \frac{\sum_{r\in Rows(T)}c\left(r,T,\Gamma \right)}{\left|Rows(T)\right|}. \end{array}$$

## 4. Construction of Directed Acyclic Graph Δ(T)

Let us consider a nonempty decision table *T* that has *n* conditional attributes $f_{1},\ldots ,f_{n}$. The Algorithm 1$A_{DAG}$ is used for the construction of a directed acyclic graph (DAG) Δ(T). Consequently, this DAG is used for the construction of optimal decision trees. Some separable subtables of the table *T* are the nodes of this DAG. We process one node during each iteration of the algorithm. We begin by the graph consisting of unprocessed one node *T* and end by processing all nodes of the graph. This algorithm was described and used in [9,10,12]. It is a special version of the more general algorithm considered in [17].
**Algorithm 1**$A_{DAG}$ (building of DAG Δ(T)).*Input*: A nonempty decision table *T* that has *n* conditional attributes $f_{1},\ldots ,f_{n}$.*Output*: Directed acyclic graph Δ(T).Build the graph consisting of one node *T* that is not tagged as processed.Check the processing of all nodes of the graph is completed or not. If yes, then the algorithm halts and returns the resulting graph as Δ(T). Otherwise, select a node (table) Θ which is yet unprocessed.Check node Θ is degenerate or not.
(a)If yes, then tag the node Θ as processed and move to step 2.(b)If no, then draw a bundle of edges from the node Θ for each $f_{i}\in E\left(\Theta \right)$. Let $E\left(\Theta ,f_{i}\right)=\left\{a_{1},\ldots ,a_{k}\right\}$. Then draw *k* edges from Θ and attach to these edges systems of equations $\left\{f_{i}=a_{1}\right\},\ldots ,\left\{f_{i}=a_{k}\right\}$. These edges enter nodes $\Theta \left\{f_{i}=a_{1}\right\},\ldots ,\Theta \left\{f_{i}=a_{k}\right\}$, respectively. In case some of the nodes $\Theta \left\{f_{i}=a_{1}\right\},\ldots ,\Theta \left\{f_{i}=a_{k}\right\}$ are not available in the graph, then add these nodes to the graph. Tag the node Θ as processed and move to step 2.


## 5. Construction of Decision Trees with Minimum Depth

Let us consider a nonempty decision table *T* that contains *n* conditional attributes $f_{1},\ldots ,f_{n}$ and k∈{1,…,5}. We can use the DAG Δ(T) to construct a decision tree $\Gamma^{\left(k\right)}\left(T\right)$ of the type *k* with the minimum depth for the decision table *T*. For this purpose, we have to construct, corresponding to each vertex Θ of Δ(T), a decision tree $\Gamma^{\left(k\right)}\left(\Theta \right)$ of the type *k* with minimum depth for the table Θ. It is necessary not only consider subtables corresponding to the nodes of Δ(T) but also empty subtable Λ of *T* as well as subtables $T_{r}$ containing only one row *r* of *T*, which are not nodes of Δ(T). The idea is to start with these special subtables as well as leaf nodes of Δ(T) that are degenerate separable subtables of *T*. In this way, we move step wise in a bottom up fashion to the table *T*.

Let us consider the case when Θ is a leaf node of Δ(T) or $\Theta =T_{r}$ for a row *r* of the table *T*, or T=Λ. If Θ is nonempty, then $\Gamma^{\left(k\right)}\left(\Theta \right)$ has only one node that is tagged by a decision which is attached to all rows of Θ. Otherwise, it is tagged with 0.

Let us consider other case when Θ is an internal node of Δ(T) and the construction of the decision tree $\Gamma^{\left(k\right)}\left(\Theta^{\prime }\right)$ is already completed for each child $\Theta^{\prime }$ of Θ. Based on these trees, a decision tree for Θ having the minimum depth can be constructed that uses decision trees of the type *k* for the subtables corresponding to the children of the root. In this tree, the root can be tagged as follows:

- By an attribute from F(T) (such decision tree can be designated as $\Gamma_{a}^{\left(k\right)}\left(\Theta \right)$).
- By a hypothesis over *T* (such decision tree can be designated as $\Gamma_{h}^{\left(k\right)}\left(\Theta \right)$).
- By a proper hypothesis over *T* (such decision tree can be designated as $\Gamma_{p}^{\left(k\right)}\left(\Theta \right)$).

The set E(Θ) is nonempty because Θ is nondegenerate. Now, three procedures for the construction of the trees $\Gamma_{a}^{\left(k\right)}\left(\Theta \right)$, $\Gamma_{h}^{\left(k\right)}\left(\Theta \right)$, and $\Gamma_{p}^{\left(k\right)}\left(\Theta \right)$ are described.

We now concentrate on a decision tree $\Gamma (f_{i})$ for the node Θ, where the root is tagged by an attribute $f_{i}\in E\left(\Theta \right)$. For each $\delta \in E(T,f_{i})$, there exists an edge that exits the root and enters the root of the decision tree $\Gamma^{\left(k\right)}\left(\Theta \left\{f_{i}=\delta \right\}\right)$. We tag this edge by the equation system $\left\{f_{i}=\delta \right\}$. It is obvious that

(1) $$h\left(\Gamma \left(f_{i}\right)\right)=1+\max\left\{h\left(\Gamma^{\left(k\right)}\left(\Theta \left\{f_{i}=\delta \right\}\right)\right):\delta \in E\left(T,f_{i}\right)\right\}.$$

One can easily prove using (1) that $\Gamma (f_{i})$ is a decision tree with the minimum depth for Θ such that the root of this tree is tagged by the attribute $f_{i}$ and it uses decision trees of the type *k* for the subtables corresponding to the children of the root.

It is obvious not to consider attributes $f_{i}\in F\left(T\right)\setminus E\left(\Theta \right)$. The reason is that for such $f_{i}$, we can find $\delta \in E(T,f_{i})$ with $\Theta \{f_{i}=\delta \}=\Theta$. Therefore, we cannot construct an optimal tree for Θ based on $f_{i}$.

**Construction of the tree**$\Gamma_{a}^{\left(k\right)}\left(\Theta \right)$. We build the set E(Θ). For any $f_{i}\in E\left(\Theta \right)$, construct the decision tree $\Gamma (f_{i})$ and choose among these trees a tree with the minimum depth. Return this tree as $\Gamma_{a}^{\left(k\right)}\left(\Theta \right)$.

Let us consider a hypothesis $H=\{f_{1}=\delta_{1},\ldots ,f_{n}=\delta_{n}\}$ over *T*. We call this hypothesis admissible for Θ and an attribute $f_{i}\in F\left(T\right)=\left\{f_{1},\ldots ,f_{n}\right\}$ if $\Theta \{f_{i}=\sigma \}\neq \Theta$ for any $\sigma \in E\left(T,f_{i}\right)\setminus \left\{\delta_{i}\right\}$. This hypothesis is not admissible for Θ and an attribute $f_{i}\in F\left(T\right)$ if and only if $|E(\Theta ,f_{i})|=1$ and $\delta_{i}\notin E\left(\Theta ,f_{i}\right)$. We call *H* admissible for Θ when we find that *H* is admissible for Θ and any attribute $f_{i}\in F\left(T\right)$.

We now describe a decision tree Γ(H) for Θ. The root of this tree is tagged by an admissible hypothesis $H=\{f_{1}=\delta_{1},\ldots ,f_{n}=\delta_{n}\}$ for Θ. For any equation system S∈A(H), there is an edge that exits the root of Γ(H) and enters the root of the tree $\Gamma^{\left(k\right)}\left(\Theta S\right)$ This edge is tagged by the equation system *S*.

It is obvious that

(2) $$h\left(\Gamma \left(H\right)\right)=1+\max\{h\left(\Gamma^{\left(k\right)}\left(\Theta S\right)\right):S\in A\left(H\right)\}.$$

One can easily prove using (2) that Γ(H) is a decision tree with the minimum depth for Θ such that the root of this tree is tagged by the hypothesis *H* and it uses decision trees of the type *k* for the subtables corresponding to the children of the root.

It is obvious not to consider hypotheses *H* that are not admissible for Θ. The reason is that for such *H*, we can find an equation system S∈A(H) with ΘS=Θ. Therefore, we cannot construct an optimal decision tree for Θ based on *H*.

**Construction of the tree**$\Gamma_{h}^{\left(k\right)}\left(\Theta \right)$. Construct a hypothesis $H_{\Theta }=\left\{f_{1}=\delta_{1},\ldots ,f_{n}=\delta_{n}\right\}$ for Θ. If $f_{i}\in F\left(T\right)\setminus E\left(\Theta \right)$, then $\delta_{i}$ is the only value from $E(\Theta ,f_{i})$. If $f_{i}\in E\left(\Theta \right)$, then $\delta_{i}$ is minimum number from $E(\Theta ,f_{i})$ for which $h\left(\Gamma^{\left(k\right)}\left(\Theta \left\{f_{i}=\delta_{i}\right\}\right)\right)=\max\left\{h\left(\Gamma^{\left(k\right)}\left(\Theta \left\{f_{i}=\sigma \right\}\right)\right):\sigma \in E\left(\Theta ,f_{i}\right)\right\}$. Return the tree $\Gamma (H_{\Theta })$ as $\Gamma_{h}^{\left(k\right)}\left(\Theta \right)$. Using (2), one can prove the correctness of this procedure.

**Construction of the tree**$\Gamma_{p}^{\left(k\right)}\left(\Theta \right)$. For each row $r=(\delta_{1},\ldots ,\delta_{n})$ of the decision table *T*, we consider a proper hypothesis $H_{r}$$=\{f_{1}=\delta_{1},\ldots ,f_{n}=\delta_{n}\}$. We inspect if $H_{r}$ is admissible for Θ. If yes, then we construct the decision tree $\Gamma (H_{r})$. We choose among the constructed trees a tree with the minimum depth. Return this tree as $\Gamma_{p}^{\left(k\right)}\left(\Theta \right)$.

Given input of a decision table *T* and k∈{1,…,5}, the following Algorithm 2$C_{h}$ builds for each node Θ of the DAG Δ(T) a decision tree $\Gamma^{\left(k\right)}\left(\Theta \right)$ of the type *k* for the table Θ having the minimum depth.
**Algorithm 2**$C_{h}$ (construction of the tree $\Gamma^{\left(k\right)}\left(T\right)$).*Input*: *T* (a nonempty decision table), Δ(T) (the directed acyclic graph for *T*), and *k* (a natural number between 1 to 5).*Output*: A decision tree $\Gamma^{\left(k\right)}\left(T\right)$.Check all nodes of the DAG Δ(T) whether there is a decision tree attached to each node. If yes, then return the tree attached to the node *T* as $\Gamma^{\left(k\right)}\left(T\right)$ and break the algorithm. If not, select a node Θ of the graph Δ(T) that does not have an attached tree. It can be either a leaf node of Δ(T) or an internal node of Δ(T) where all children are tagged with trees.If Θ is a leaf node, then attach to it the decision tree $\Gamma^{\left(k\right)}\left(\Theta \right)$ that have only a single node. This node is tagged with the decision attached to all rows of Θ. Move to step 1.If Θ is not a leaf node, then do the following according to the value *k*:When k=1, construct the tree $\Gamma_{a}^{\left(1\right)}\left(\Theta \right)$ and attach it to Θ as the tree $\Gamma^{\left(1\right)}\left(\Theta \right)$.When k=2, construct the tree $\Gamma_{h}^{\left(2\right)}\left(\Theta \right)$ and attach it to Θ as the tree $\Gamma^{\left(2\right)}\left(\Theta \right)$.When k=3, construct the trees $\Gamma_{a}^{\left(3\right)}\left(\Theta \right)$ and $\Gamma_{h}^{\left(3\right)}\left(\Theta \right)$, choose between them a tree with the minimum depth and attach it to Θ as the tree $\Gamma^{\left(3\right)}\left(\Theta \right)$.When k=4, construct the tree $\Gamma_{p}^{\left(4\right)}\left(\Theta \right)$ and attach it to Θ as the tree $\Gamma^{\left(4\right)}\left(\Theta \right)$.When k=5, construct the trees $\Gamma_{a}^{\left(5\right)}\left(\Theta \right)$ and $\Gamma_{p}^{\left(5\right)}\left(\Theta \right)$, choose between them a tree with the minimum depth and attach it to Θ as the tree $\Gamma^{\left(5\right)}\left(\Theta \right)$.Move to step 1.

Let *T* be a decision table and k∈{1,…,5}. We use the following notation: $l_{h}^{\left(k\right)}\left(T\right)=l\left(T,\Gamma^{\left(k\right)}\left(T\right)\right)$ and $c_{h}^{\left(k\right)}\left(T\right)=c\left(T,\Gamma^{\left(k\right)}\left(T\right)\right)$.

## 6. Construction of Decision Trees Containing Minimum Number of Internal Nodes

Let us consider a nonempty decision table *T* that contains *n* conditional attributes $f_{1},\ldots ,f_{n}$ and k∈{1,…,5}. We can use the DAG Δ(T) to construct a decision tree $G^{\left(k\right)}\left(T\right)$ of the type *k* with the minimum number of internal nodes for the decision table *T*. To construct the tree $G^{\left(k\right)}\left(T\right)$, for each node Θ of the DAG Δ(T), we construct a decision tree $G^{\left(k\right)}\left(\Theta \right)$ of the type *k* with the minimum number of internal nodes for the decision table Θ. It is necessary to not only consider the subtables corresponding to the nodes of Δ(T) but also the empty subtable Λ of *T* as well as the subtables $T_{r}$ containing only one row *r* of *T* which are not nodes of Δ(T). The idea is to start with these special subtables as well as leaf nodes of Δ(T) that are degenerate separable subtables of *T*. In this way, we move step wise in a bottom up fashion to the table *T*.

Let us consider the case when Θ is a leaf node of Δ(T) or $\Theta =T_{r}$ for a row *r* of the table *T*, or T=Λ. If Θ is nonempty, then the decision tree $G^{\left(k\right)}\left(\Theta \right)$ has only one node that is tagged by a decision which is attached to all rows of Θ. Otherwise, it is tagged with 0.

Let us consider another case when Θ is an internal node of Δ(T) such that the construction of the decision tree $G^{\left(k\right)}\left(\Theta^{\prime }\right)$ is already completed for each child $\Theta^{\prime }$ of Θ. Based on these trees, a decision tree containing the minimum number of internal nodes for Θ can be constructed that uses decision trees of the type *k* for the subtables corresponding to the children of the root. In this tree, the root can be tagged as follows:

- By an attribute from F(T) (such decision tree can be designated as $G_{a}^{\left(k\right)}\left(\Theta \right)$).
- By a hypothesis over *T* (such decision tree can be designated as $G_{h}^{\left(k\right)}\left(\Theta \right)$).
- By a proper hypothesis over *T* (such decision tree can be designated as $G_{p}^{\left(k\right)}\left(\Theta \right)$).

The set E(Θ) is nonempty because Θ is nondegenerate. Now, three procedures for the construction of the trees $G_{a}^{\left(k\right)}\left(\Theta \right)$, $G_{h}^{\left(k\right)}\left(\Theta \right)$, and $G_{p}^{\left(k\right)}\left(\Theta \right)$ are described.

We now concentrate on a decision tree $G(f_{i})$ for the node Θ where the root is tagged by an attribute $f_{i}\in E\left(\Theta \right)$. For each $\delta \in E(T,f_{i})$, there is an edge that exits the root and enters the root of the decision tree $G^{\left(k\right)}\left(\Theta \left\{f_{i}=\delta \right\}\right)$. We tag this edge by the equation system $\left\{f_{i}=\delta \right\}$. It is obvious that

(3) $$L_{w}\left(G\left(f_{i}\right)\right)=1+\sum_{\delta \in E(T,f_{i})}L_{w}\left(G^{\left(k\right)}\left(\Theta \left\{f_{i}=\delta \right\}\right)\right).$$

One can easily prove using (3) that $G(f_{i})$ is a decision tree with the minimum number of internal nodes for Θ such that the root of the tree is tagged by the attribute $f_{i}$ and it uses decision trees of the type *k* for the subtables corresponding to the children of the root.

It is obvious not to consider attributes $f_{i}\in F\left(T\right)\setminus E\left(\Theta \right)$. The reason is that for such $f_{i}$, we can find $\delta \in E(T,f_{i})$ with $\Theta \{f_{i}=\delta \}=\Theta$. Therefore, we cannot construct an optimal tree for Θ based on $f_{i}$.

**Construction of the tree**$G_{a}^{\left(k\right)}\left(\Theta \right)$. We build the set of attributes E(Θ). For any $f_{i}\in E\left(\Theta \right)$, construct the decision tree $G(f_{i})$ and choose among these trees a tree with the minimum number of internal nodes. We return this tree as $G_{a}^{\left(k\right)}\left(\Theta \right)$.

We now describe a decision tree G(H) for Θ. The root of this tree is tagged by an admissible hypothesis $H=\{f_{1}=\delta_{1},\ldots ,f_{n}=\delta_{n}\}$ for Θ. For any equation system S∈A(H), there is an edge that exits the root of G(H) and enters the root of the tree $G^{\left(k\right)}\left(\Theta S\right)$. This edge is tagged by the equation system *S*. It is obvious that

(4) $$L_{w}\left(G\left(H\right)\right)=1+\sum_{S\in A(H)}L_{w}\left(G^{\left(k\right)}\left(\Theta S\right)\right).$$

One can easily prove using (4) that G(H) is a decision tree with the minimum number of internal nodes for Θ such that the root of the tree is tagged by the hypothesis *H* and it uses decision trees of the type *k* for the subtables corresponding to the children of the root.

It is obvious not to consider hypotheses *H* that are not admissible for Θ. The reason is that for such *H*, we can find an equation system S∈A(H) with ΘS=Θ. Therefore, we cannot construct an optimal decision tree for Θ based on such *H*.

**Construction of the tree**$G_{h}^{\left(k\right)}\left(\Theta \right)$. We construct a hypothesis $H_{\Theta }=\left\{f_{1}=\delta_{1},\ldots ,f_{n}=\delta_{n}\right\}$ for Θ. If $f_{i}\notin E\left(\Theta \right)$, then $\delta_{i}$ is the only value in $E(\Theta ,f_{i})$. Let $f_{i}\in E\left(\Theta \right)$. Then $\delta_{i}$ is the minimum number from $E(\Theta ,f_{i})$ such that $L_{w}\left(G^{\left(k\right)}\left(\Theta \left\{f_{i}=\delta_{i}\right\}\right)\right)=\max\left\{L_{w}\left(G^{\left(k\right)}\left(\Theta \left\{f_{i}=\sigma \right\}\right)\right):\sigma \in E\left(\Theta ,f_{i}\right)\right\}$. Obviously, $H_{\Theta }$ is admissible for Θ. Return the tree $G(H_{\Theta })$ as $G_{h}^{\left(k\right)}\left(\Theta \right)$. Using (4), one can prove the correctness of this procedure.

**Construction of the tree**$G_{p}^{\left(k\right)}\left(\Theta \right)$. For each row $r=(\delta_{1},\ldots ,\delta_{n})$ of the decision table *T*, let us consider a proper hypothesis $H_{r}$ $=\{f_{1}=\delta_{1},\ldots ,f_{n}=\delta_{n}\}$. We inspect if $H_{r}$ is admissible for Θ. If yes, then we construct the decision tree $G(H_{r})$. We choose among the constructed trees a tree with the minimum number of internal nodes. Return this tree as $G_{p}^{\left(k\right)}\left(\Theta \right)$.

Given input of a decision table *T* and k∈{1,…,5}, the following Algorithm 3$C_{L_{w}}$ builds for each node Θ of the DAG Δ(T) a decision tree $G^{\left(k\right)}\left(\Theta \right)$ of the type *k* for the table Θ having the minimum number of internal nodes.
**Algorithm 3**$C_{L_{w}}$ (construction of the tree $G^{\left(k\right)}\left(T\right)$).*Input*: *T* (a nonempty decision table), Δ(T) (the directed acyclic graph for *T*), and *k* (a natural number between 1 to 5).*Output*: A decision tree $G^{\left(k\right)}\left(T\right)$.Check all nodes of the DAG Δ(T) whether there is a decision tree attached to each node. If yes, then return the tree attached to the node *T* as $G^{\left(k\right)}\left(T\right)$ and break the algorithm. If not, select a node Θ of the graph Δ(T) that does not have an attached tree. It can be either a leaf node of Δ(T) or an internal node of Δ(T) where all children are tagged with trees.If Θ is a leaf node, then attach to it the decision tree $G^{\left(k\right)}\left(\Theta \right)$ that have only a single node. This node is tagged with the decision attached to all rows of Θ. Move to step 1.If Θ is not a leaf node, then do the following according to the value *k*:
When k=1, construct the tree $G_{a}^{\left(1\right)}\left(\Theta \right)$ and attach it to Θ as the tree $G^{\left(1\right)}\left(\Theta \right)$.When k=2, construct the tree $G_{h}^{\left(2\right)}\left(\Theta \right)$ and attach it to Θ as the tree $G^{\left(2\right)}\left(\Theta \right)$.When k=3, construct the trees $G_{a}^{\left(3\right)}\left(\Theta \right)$ and $G_{h}^{\left(3\right)}\left(\Theta \right)$, choose between them a tree with the minimum number of internal nodes and attach it to Θ as the tree $G^{\left(3\right)}\left(\Theta \right)$.When k=4, construct the tree $G_{p}^{\left(4\right)}\left(\Theta \right)$ and attach it to Θ as the tree $G^{\left(4\right)}\left(\Theta \right)$.When k=5, construct the trees $G_{a}^{\left(5\right)}\left(\Theta \right)$ and $G_{p}^{\left(5\right)}\left(\Theta \right)$, choose between them a tree with the minimum number of internal nodes and attach it to Θ as the tree $G^{\left(5\right)}\left(\Theta \right)$.
Move to step 1.

Let *T* be a decision table and k∈{1,…,5}. We use the following notation: $l_{L_{w}}^{\left(k\right)}\left(T\right)=l\left(T,G^{\left(k\right)}\left(T\right)\right)$ and $c_{L_{w}}^{\left(k\right)}\left(T\right)=c\left(T,G^{\left(k\right)}\left(T\right)\right)$.

## 7. Experimental Results and Discussion

In this section, we describe the results of the experiments. First, we accomplished the experiments on eight decision tables from the UCI ML Repository [16]. We give the description of these tables in Table 1 where we show first the name of the table (Name), then number of rows (#Rows) and the number of attributes (#Attrs). We arranged the decision tables in Table 1 based on the number of rows. For each of these tables, we built an optimal decision tree of each of five possible types for each of the two possible cost functions. From these trees, we derive decision rules and study their coverage and length.

Next, we experimented with 100 Boolean functions having *n* variables (n=3,…,6) which are generated randomly. Let *f* be such a Boolean function with *n* variables $x_{1},\ldots ,x_{n}$. We can map it to a decision table $T_{f}$ having *n* attributes $x_{1},\ldots ,x_{n}$. This table has $2^{n}$ rows corresponding to all possible *n*-tuples of variable values. We label each row with the decision that is the value of the function *f* for the considered row. The decision trees for the table $T_{f}$ are interpreted as decision trees that compute the function *f*.

For each of tables representing the generated Boolean functions, we build an optimal decision tree of each of five possible types for each of the two possible cost functions. From these trees, we derive decision rules and study their coverage and length.

### 7.1. Decision Trees with Minimum Depth

The results of experiments based on eight decision tables from [16] and decision trees optimal relative to the depth are represented in Table 2 and Table 3. The first column of Table 2 contains the name of the considered decision table *T*. The last five columns contain values $l_{h}^{\left(1\right)}\left(T\right),\ldots ,l_{h}^{\left(5\right)}\left(T\right)$ (minimum values for each decision table are in bold).

The first column of Table 3 contains the name of the considered decision table *T*. The last five columns contain values $c_{h}^{\left(1\right)}\left(T\right),\ldots ,c_{h}^{\left(5\right)}\left(T\right)$ (maximum values for each decision table are in bold).

The results of experiments based on Boolean functions and decision trees optimal relative to the depth are represented in Table 4 and Table 5. The first column of Table 4 contains the number *n* of variables in the considered Boolean functions. The last five columns contain information about values $l_{h}^{\left(1\right)},\ldots ,l_{h}^{\left(5\right)}$ in the format ${}_{\min}{\mathrm{Avg}}_{\max}$ (minimum values of Avg for each *n* are in bold).

The first column of Table 5 contains the number *n* of variables in the considered Boolean functions. The last five columns contain information about values $c_{h}^{\left(1\right)},\ldots ,c_{h}^{\left(5\right)}$ in the format ${}_{\min}{\mathrm{Avg}}_{\max}$ (maximum values of Avg for each *n* are in bold).

### 7.2. Decision Trees Containing Minimum Number of Internal Nodes

We present the results based on the decision tables from [16] and decision trees optimal relative to the number of internal nodes in Table 6 and Table 7. The first column of Table 6 contains the name of the considered decision table *T*. The last five columns contain values $l_{L_{w}}^{\left(1\right)}\left(T\right),\ldots ,l_{L_{w}}^{\left(5\right)}\left(T\right)$ (minimum values for each decision table are in bold).

The first column of Table 7 contains the name of the considered decision table *T*. The last five columns contain values $c_{L_{w}}^{\left(1\right)}\left(T\right),\ldots ,c_{L_{w}}^{\left(5\right)}\left(T\right)$ (maximum values for each decision table are in bold).

The results of experiments based on Boolean functions and decision trees optimal relative to the number of internal nodes are represented in Table 8 and Table 9. The first column of Table 8 contains the number *n* of variables in the considered Boolean functions. The last five columns contain information about values $l_{L_{w}}^{\left(1\right)},\ldots ,l_{L_{w}}^{\left(5\right)}$ in the format ${}_{\min}{\mathrm{Avg}}_{\max}$ (minimum values of Avg for each *n* are in bold).

The first column of Table 9 contains the number of variables *n* in the considered Boolean functions. The last five columns contain information about values $c_{L_{w}}^{\left(1\right)},\ldots ,c_{L_{w}}^{\left(5\right)}$ in the format ${}_{\min}{\mathrm{Avg}}_{\max}$ (maximum values of Avg for each *n* are in bold).

### 7.3. Analysis of Experimental Results

The experimental results show that the decision rules derived from decision trees with hypotheses in many cases are better than the ones derived from conventional decision trees. In particular, in the case of decision trees with the minimum depth, for each row in Table 2, Table 3, Table 4 and Table 5, the results for type 2 decision trees are better than the results for type 1 decision trees. In the case of decision trees with a minimum number of internal nodes, for each row of Table 6, Table 7, Table 8 and Table 9 (with the exception of the row zoo-data in Table 7) there is a number k∈{2,…,5} such that the results for type *k* decision trees are superior compared to the results for type 1 decision trees.

Note that for the decision trees with the minimum depth, for each decision table from [16] considered in this paper, the best results related to the length and the coverage among decision trees of types {2,…,5} are close to the optimal ones obtained in [18] with the help of dynamic programming algorithms for the construction of optimal decision rules. Results for the decision trees of the type 1 are, generally, far from the optimal ones.

From the obtained experimental results, it follows that the decision rules derived from optimal decision trees with hypotheses are more preferable as a tool for the representation of information than the decision rules derived from optimal conventional decision trees.

## 8. Conclusions

In this paper, we studied modified decision trees that use two types of queries. We constructed optimal trees relative to two cost functions for a number of known datasets from the UCI Machine Learning Repository and randomly generated Boolean functions, and compared the length as well as coverage of decision rules extracted from the constructed decision trees. The experimental results confirmed that the decision rules derived from the decision trees with hypotheses in many cases are better than the ones derived from conventional decision trees.

## Figures and Tables

**Table 1:** Description of decision tables from [16] which were used in experiments.

Name	#Rows	#Attrs
soybean-small	47	36
zoo-data	59	17
hayes-roth-data	69	5
breast-cancer	266	10
balance-scale	625	5
tic-tac-toe	958	10
cars	1728	7
nursery	12,960	9

**Table 2:** Results for decision tables from [16]: length of decision rules derived from decision trees with minimum depth.

Decision Table *T*	$l_{h}^{\left(1\right)}\left(T\right)$	$l_{h}^{\left(2\right)}\left(T\right)$	$l_{h}^{\left(3\right)}\left(T\right)$	$l_{h}^{\left(4\right)}\left(T\right)$	$l_{h}^{\left(5\right)}\left(T\right)$
soybean-small	1.89	**1.00**	1.89	1.55	1.89
zoo-data	3.69	**1.56**	2.42	2.17	3.24
hayes-roth-data	2.83	2.22	**2.16**	2.32	2.35
breast-cancer	3.61	**2.68**	2.71	2.70	2.78
balance-scale	3.60	**3.20**	**3.20**	3.21	**3.20**
tic-tac-toe	5.09	**3.04**	3.40	3.24	3.14
cars	3.72	**2.44**	2.48	3.07	3.02
nursery	5.78	3.16	4.53	**3.12**	4.50
Average	3.78	2.41	2.85	2.67	3.01

**Table 3:** Results for decision tables from [16]: coverage of decision rules derived from decision trees with minimum depth.

Decision Table *T*	$c_{h}^{\left(1\right)}\left(T\right)$	$c_{h}^{\left(2\right)}\left(T\right)$	$c_{h}^{\left(3\right)}\left(T\right)$	$c_{h}^{\left(4\right)}\left(T\right)$	$c_{h}^{\left(5\right)}\left(T\right)$
soybean-small	3.47	**12.53**	3.47	10.62	3.47
zoo-data	7.88	10.78	9.80	**10.86**	6.46
hayes-roth-data	3.46	6.20	**6.49**	5.48	5.45
breast-cancer	4.98	9.30	8.36	**9.38**	6.90
balance-scale	2.60	**4.19**	4.18	4.16	**4.19**
tic-tac-toe	8.38	**66.01**	27.23	56.64	61.45
cars	197.60	**332.76**	330.35	97.20	99.42
nursery	29.33	1524.04	304.71	**1530.50**	246.14
Average	32.21	245.73	86.82	215.60	54.18

**Table 4:** Results for Boolean functions: length of decision rules derived from decision trees with minimum depth.

Number of Variables *n*	$l_{h}^{\left(1\right)}$	$l_{h}^{\left(2\right)}$	$l_{h}^{\left(3\right)}$	$l_{h}^{\left(4\right)}$	$l_{h}^{\left(5\right)}$
3	${}_{1.50}2.20_{2.75}$	${}_{1.25}2.05_{2.63}$	${}_{1.25}1.99_{2.50}$	${}_{1.25}2.08_{2.63}$	${}_{1.25}2.01_{2.50}$
4	${}_{1.88}3.18_{3.75}$	${}_{1.63}2.92_{3.50}$	${}_{1.63}2.87_{3.50}$	${}_{1.63}2.91_{3.50}$	${}_{1.63}2.94_{3.50}$
5	${}_{3.44}4.09_{4.63}$	${}_{3.00}3.64_{4.22}$	${}_{2.97}3.60_{4.06}$	${}_{3.13}3.66_{4.13}$	${}_{3.09}3.70_{4.19}$
6	${}_{4.78}5.14_{5.47}$	${}_{3.98}4.36_{4.77}$	${}_{3.98}4.41_{4.75}$	${}_{3.97}4.35_{4.70}$	${}_{4.03}4.46_{4.78}$

**Table 5:** Results for Boolean functions: coverage of decision rules derived from decision trees with minimum depth.

Number of Variables *n*	$c_{h}^{\left(1\right)}$	$c_{h}^{\left(2\right)}$	$c_{h}^{\left(3\right)}$	$c_{h}^{\left(4\right)}$	$c_{h}^{\left(5\right)}$
3	${}_{1.25}1.94_{3.00}$	${}_{1.38}2.22_{3.63}$	${}_{1.50}2.21_{3.63}$	${}_{1.38}2.14_{3.63}$	${}_{1.50}2.17_{3.63}$
4	${}_{1.25}1.99_{5.38}$	${}_{1.50}2.57_{6.44}$	${}_{1.50}2.52_{6.44}$	${}_{1.50}2.53_{6.44}$	${}_{1.50}2.36_{6.44}$
5	${}_{1.38}2.10_{3.69}$	${}_{2.03}3.03_{4.56}$	${}_{2.06}2.98_{4.97}$	${}_{2.03}2.93_{4.69}$	${}_{1.81}2.76_{4.66}$
6	${}_{1.59}2.03_{2.84}$	${}_{2.69}3.55_{4.81}$	${}_{2.58}3.37_{4.64}$	${}_{2.72}3.53_{4.70}$	${}_{2.53}3.24_{4.69}$

**Table 6:** Results for decision tables from [16]: length of decision rules derived from decision trees with minimum number of internal nodes.

Decision Table *T*	$l_{L_{w}}^{\left(1\right)}\left(T\right)$	$l_{L_{w}}^{\left(2\right)}\left(T\right)$	$l_{L_{w}}^{\left(3\right)}\left(T\right)$	$l_{L_{w}}^{\left(4\right)}\left(T\right)$	$l_{L_{w}}^{\left(5\right)}\left(T\right)$
soybean-small	1.34	**1.00**	1.34	1.51	1.34
zoo-data	3.05	**1.69**	3.05	2.39	3.05
hayes-roth-data	2.64	**2.22**	2.61	2.23	2.61
breast-cancer	4.98	**2.72**	5.30	2.73	5.27
balance-scale	3.55	**3.20**	3.53	**3.20**	3.53
tic-tac-toe	4.45	3.35	4.41	**3.15**	4.45
cars	2.97	**2.49**	2.96	**2.49**	2.96
nursery	3.77	**3.19**	3.77	**3.19**	3.77
Average	3.34	2.48	3.37	2.61	3.37

**Table 7:** Results for decision tables from [16]: coverage of decision rules derived from decision trees with minimum number of internal nodes.

Decision Table *T*	$c_{L_{w}}^{\left(1\right)}\left(T\right)$	$c_{L_{w}}^{\left(2\right)}\left(T\right)$	$c_{L_{w}}^{\left(3\right)}\left(T\right)$	$c_{L_{w}}^{\left(4\right)}\left(T\right)$	$c_{L_{w}}^{\left(5\right)}\left(T\right)$
soybean-small	11.51	**12.53**	11.51	9.81	11.51
zoo-data	**10.69**	10.68	**10.69**	10.63	**10.69**
hayes-roth-data	3.84	**6.20**	3.87	**6.20**	3.87
breast-cancer	2.73	8.96	3.05	**9.06**	3.15
balance-scale	2.79	**4.21**	2.88	**4.21**	2.88
tic-tac-toe	22.49	30.19	23.50	**56.50**	22.69
cars	237.33	**332.46**	237.37	**332.46**	237.37
nursery	1471.45	**1527.95**	1471.47	**1527.95**	1471.47
Average	220.35	241.65	220.54	244.60	220.45

**Table 8:** Results for Boolean functions: length of decision rules derived from decision trees with minimum number of internal nodes.

Number of Variables *n*	$l_{L_{w}}^{\left(1\right)}$	$l_{L_{w}}^{\left(2\right)}$	$l_{L_{w}}^{\left(3\right)}$	$l_{L_{w}}^{\left(4\right)}$	$l_{L_{w}}^{\left(5\right)}$
3	${}_{1.50}2.07_{2.75}$	${}_{1.25}2.06_{2.63}$	${}_{1.25}1.94_{2.50}$	${}_{1.25}2.06_{2.63}$	${}_{1.25}1.94_{2.50}$
4	${}_{1.88}2.90_{3.50}$	${}_{1.63}2.94_{3.50}$	${}_{1.81}2.79_{3.50}$	${}_{1.63}2.94_{3.50}$	${}_{1.81}2.79_{3.50}$
5	${}_{3.13}3.75_{4.19}$	${}_{3.00}3.77_{4.31}$	${}_{3.13}3.69_{4.25}$	${}_{3.00}3.77_{4.31}$	${}_{3.13}3.69_{4.25}$
6	${}_{4.28}4.69_{5.06}$	${}_{4.13}4.69_{5.19}$	${}_{4.25}4.61_{4.98}$	${}_{4.13}4.69_{5.19}$	${}_{4.25}4.61_{4.98}$

**Table 9:** Results for Boolean functions: coverage of decision rules derived from decision trees with minimum number of internal nodes.

Number of Variables *n*	$c_{L_{w}}^{\left(1\right)}$	$c_{L_{w}}^{\left(2\right)}$	$c_{L_{w}}^{\left(3\right)}$	$c_{L_{w}}^{\left(4\right)}$	$c_{L_{w}}^{\left(5\right)}$
3	${}_{1.25}2.14_{3.00}$	${}_{1.38}2.22_{3.63}$	${}_{1.50}2.27_{3.63}$	${}_{1.38}2.22_{3.63}$	${}_{1.50}2.27_{3.63}$
4	${}_{1.63}2.51_{5.38}$	${}_{1.50}2.56_{6.44}$	${}_{1.50}2.63_{5.44}$	${}_{1.50}2.56_{6.44}$	${}_{1.50}2.63_{5.44}$
5	${}_{1.88}2.79_{4.19}$	${}_{1.94}2.91_{4.59}$	${}_{1.75}2.82_{4.34}$	${}_{1.94}2.91_{4.59}$	${}_{1.75}2.82_{4.34}$
6	${}_{2.16}2.88_{4.09}$	${}_{2.09}3.16_{4.58}$	${}_{2.27}2.99_{4.11}$	${}_{2.09}3.16_{4.58}$	${}_{2.27}2.99_{4.11}$

## Data Availability

Data are available in a publicly accessible repository that does not issue DOIs. Publicly available datasets were analyzed in this study. These data can be found here: http://archive.ics.uci.edu/ml (accessed on 12 April 2017).
